# Ausbildung im Zeitalter der Digitalisierung – Evaluation des digitalen Münsteraner Dermatopathologiekurses

**DOI:** 10.1007/s00105-025-05516-x

**Published:** 2025-05-23

**Authors:** Paul Schmidle, Dieter Metze, Ole Hätscher, Stephan Alexander Braun

**Affiliations:** 1 Klinik für Hautkrankheiten, Universitätsklinikum Münster, Von-Esmarch-Str. 58, 48149 Münster, Deutschland; 2 Institut für Ausbildung und Studienangelegenheiten der Medizinischen Fakultät, Universität Münster, Münster, Deutschland; 3 Institut für Psychologie, Universität Münster, Münster, Deutschland; 4 Klinik für Dermatologie, Medizinische Fakultät, Heinrich-Heine-Universität, Düsseldorf, Deutschland

**Keywords:** Lehre, Ausbildung, Dermatopathologie, Pathologie, Histologie, Teaching, Training, Dermatopathology, Pathology, Histology

## Abstract

**Hintergrund:**

Die Dermatopathologie ist ein wichtiger Baustein in der dermatologischen Diagnostik. In Münster werden seit über 20 Jahren Fortbildungskurse angeboten, die eine strukturierte Einführung in die Dermatopathologie bieten. Diese Kurse werden seit 2021 in einem digitalen Format angeboten.

**Ziel der Arbeit:**

Es erfolgen (i) die Präsentation des neuen digitalen Kursformats, (ii) systematische Auswertung der Evaluation der Onlinekurse durch die Teilnehmenden sowie (iii) Diskussion allgemeiner Vor- und Nachteile einer zunehmend digitaler werdenden Ausbildung in der Dermatopathologie.

**Material und Methoden:**

Von 2021 bis 2024 wurden die jährlich stattgefundenen Onlinekurse von den Teilnehmenden mittels eines standardisierten Fragebogens evaluiert. Es wurden Fragen zu Fachbezeichnung, Ausbildungsstand, Herkunftsland, technischer Umsetzung des digitalen Formats und Inhalt des Kurses gestellt. Die Antworten wurden statistisch ausgewertet.

**Ergebnisse:**

Die Umstellung der Kurse in ein digitales Format wurde insgesamt sehr positiv bewertet. Mit der technischen Umsetzung hatten die Teilnehmenden keine Probleme. Die Teilnehmenden wünschen in Zukunft noch mehr digitale Lehrangebote in der Dermatopathologie und bevorzugen digitale Kursangebote gegenüber analogen.

**Diskussion:**

Ergänzenden digitalen Lehrformaten gehört auch in der Dermatopathologie die Zukunft. Dass diese bereits heute technisch gut umsetzbar sind und von Teilnehmenden durchweg positiv evaluiert werden, zeigt unser Beispiel. Reine Onlineveranstaltungen haben jedoch auch Nachteile, insbesondere durch den Wegfall des persönlichen Austauschs. Eine Kombination aus Präsenzveranstaltungen und digitalen Formaten scheint für die Zukunft der beste Weg zu sein.

## Abstract (en)

**Background:**

Dermatopathology is an important component in diagnosing dermatological diseases. For more than 20 years, training courses have been offered in Münster to provide structured insight into dermatopathology. Since 2021 these courses have been offered in a digital format.

**Objectives:**

Goals were (i) to present the new digital course format, (ii) to systematically analyze the evaluation of the online courses by the participants and (iii) to discuss the general advantages and disadvantages of an increasingly digital training in dermatopathology.

**Materials and methods:**

From 2021–2024, annual online courses were evaluated by participants using a standardized questionnaire. Questions were asked about specialist title, level of training, country of origin, technical implementation of the digital format, and course content. Responses were statistically analyzed.

**Results:**

The conversion of the courses to a digital format was rated very positively overall. Participants had no problems with the technical implementation. The participants would like to see more digital courses in dermatopathology in the future and even prefer digital courses to analogue ones.

**Conclusion:**

The future also belongs to complementary digital teaching formats in dermatopathology. Our example shows that these are already technically feasible and have been consistently positively evaluated by participants. However, purely online events also have disadvantages, particularly due to the lack of personal interaction. A combination of various face-to-face and digital formats seems to be the best way forward.

Die Dermatopathologie ist nach wie vor ein wichtiger Baustein in der diagnostischen Kette dermatologischer Erkrankungen – egal ob Tumor- oder entzündliche Erkrankung. Viele Diagnosen können erst durch die Verknüpfung des makroskopischen und mikroskopischen Befundes gestellt werden. Ein histologisches Basiswissen für die klinisch-pathologische Korrelation ist demnach auch für rein klinisch arbeitende Dermatologen von großem Vorteil [1–6]. Dies spiegelt auch die aktuelle Weiterbildungsordnung für Haut- und Geschlechtskrankheiten wider, in der die Teilnahme an mindestens 50 dermatopathologischen Demonstrationen und Konferenzen gefordert wird [7].

Leider schwinden jedoch die Möglichkeiten, sich an den Ausbildungsstätten intensiver mit der Histologie von Hautkrankheiten zu beschäftigen und mit Dermatopathologen in den engen Austausch zu kommen. Junge Kollegen berichten, dass ihnen hierfür schlichtweg die Zeit fehle. Zusätzlich ist die Anzahl an Ausbildungsplätzen begrenzt. Die Situation wird sich mit der Berentung der Baby-Boomer-Jahrgänge noch weiter verschlechtern – ein Phänomen, mit dem auch die Pathologie zu kämpfen hat [8]. Es stellt sich demnach die Frage, wie in Zukunft dermatopathologisches Wissen überhaupt noch vermittelt werden soll [9].

Seit 2001 werden in Münster regelmäßig ein Einführungs- und Mikroskopierkurs zum Thema Dermatopathologie angeboten. Ziel des Einführungskurses „Von der Biopsie zur Diagnose“ ist die Vermittlung histologischen Grundwissens für die klinisch-pathologische Korrelation. Es wird auf die Fallstricke der histologischen Diagnostik aufmerksam gemacht, und das Interesse an der Dermatopathologie soll geweckt werden. Der Einführungskurs ist für klinisch arbeitende Dermatologen, um die im Facharztkatalog geforderte Indikation und Interpretation gebietsbezogener histologischer und molekularbiologischer Untersuchungen [7] thematisch abzudecken. Die Demonstration histologischer Bilder dient dabei primär als ergänzende, beispielhafte, konzeptuelle Demonstration ausgewählter Krankheitsbilder. Im Mikroskopierkurs hingegen wird unter strukturierter Anleitung tiefer in die histologische Diagnostik eingestiegen. Der Fokus liegt hier auf der Musteranalyse von entzündlichen Dermatosen und der Hauttumordiagnostik [10]. Dabei soll Verständnis für das Konzept der „lives of lesions“ [11] geschaffen, differenzialdiagnostische Überlegungen angestellt und die gezielte Nutzung von Immunhistologie diskutiert und geübt werden.

Beide Kurse wurden über mehr als 2 Jahrzehnte als Präsenzveranstaltung ausgerichtet und Präparate auf Glasobjektträgern am Lichtmikroskop mikroskopiert. Die COVID-19-Pandemie hat uns jedoch gezwungen zu überlegen, wie das Kursangebot in den digitalen Raum verschoben werden könnte. Glasobjektträger können heutzutage über spezielle Scanner zwar einfach digitalisiert werden, aber diese digitalisierten Präparate, die große Mengen an Speicherplatz benötigen [12], einer größeren Kursteilnehmendenzahl online zur Verfügung zu stellen, ist eine technische Herausforderung.

Seit 2021 werden die Dermatopathologiekurse aus Münster digital angeboten und evaluiert. Insgesamt fanden bisher 5 Kurse online statt. In dieser Arbeit werden die Ergebnisse der Evaluation des neuen Formates der Teilnehmenden präsentiert und diskutiert. Zusätzlich wird die technische Lösung des digitalen Mikroskopierkurses vorgestellt und allgemeine Vor- und Nachteile einer zunehmend digitaler werdenden Ausbildung werden beleuchtet.

## Material und Methoden

### Digitalisierung der Kurspräparate und Nutzung der Online-Lehrplattform SmartZOOM®

**Abb. 1:**
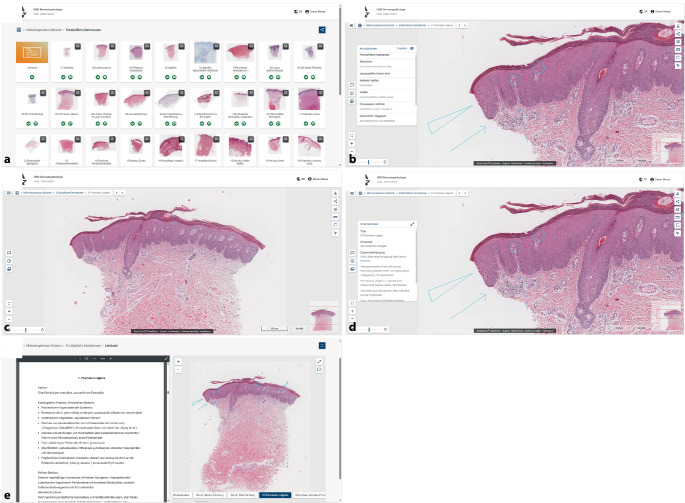
Übersichtlich geordneter Präparatekasten (**a**), Detailansicht der Präparate ohne (**b**) und mit Annotationen (**c**) sowie zusätzliche Kommentar- (**d**) und Lehrbuchfunktion (**e**)

Die Glasobjektträger der Kurspräparate für den Einführungs- und Mikroskopierkurs wurden zunächst mittels Whole Slide Scanner (Hamamatsu NanoZoomer S360 MD, Hamamatsu City, Japan) digitalisiert und als sog. Whole Slide Images (WSI) abgespeichert. Die WSI sind mehrere Gigabyte groß, und können somit nicht einfach als Download zur Verfügung gestellt werden. Ferner benötigt es zur Betrachtung der WSI einer speziellen Software. Wir entschieden uns deshalb, die gescannten Kurspräparate Cloud-basiert online zur Verfügung zu stellen. Dies ermöglicht es, WSI schnell über jeden beliebigen Internetbrowser abzurufen, ohne dass dafür eine spezielle Software installiert werden muss. Es gibt bereits mehrere Anbieter Cloud-basierter Lösungen wie beispielsweise PathPresenter® (PathPresenter Corporation, Montville, New Jersey, USA) oder smartZOOM®classROOM (Smart In Media AG, Köln, Deutschland). Wir entschieden uns für die Nutzung von smartZOOM®classROOM. Die Plattform ist browserunabhängig über einen Link aufrufbar, und der Zugang kann über ein Passwort geschützt werden [13]. Prinzipiell kann die Plattform über jedes internetfähige Endgerät aufgerufen werden, solange eine schnelle, stabile Internetverbindung besteht. Nach unseren Erfahrungen können die Präparate am besten an einem großen Bildschirm und mit einer Maus mit Scrollrad betrachtet werden. Auf der Plattform können nicht nur Präparate, sondern auch weitere Lehrmaterialien wie das Kursskript und der Programmablauf abgelegt werden. Die Kursteilnehmenden können nach Anmeldung frei durch die Plattform und die einzelnen Präparate navigieren (Abb. 1a, b). Einen großen Vorteil bietet die Möglichkeit, innerhalb der Präparate besonders relevante Strukturen zu markieren (Abb. 1c). Über die Festlegung einer Reihenfolge der Annotationen kann systematisch durch das Präparat geführt werden, analog zu einer Live-Demonstration am Lichtmikroskop (Abb. 1c). Zusätzlich zu den Annotationen können weitere Informationen und Kommentare zu den Fällen abgelegt werden, die in einem separaten Fenster ein- und ausgeblendet werden (Abb. 1d). Im Lehrbuchmodus besteht die Möglichkeit, sich das Kursskript parallel zu den annotierten Präparaten auf einem Bildschirm anzeigen zu lassen oder direkte Verlinkungen beispielsweise zu klinischen Bildern zu hinterlegen (Abb. 1e).

### Kursformat

Die Kurse wurden als Videokonferenz mit ZOOM® (ZOOM Communications, San Jose, Kalifornien, USA) durchgeführt. Im Gegensatz zu anderen Anbietern war bei ZOOM® die ruckelfreie Übertragung der Bilddaten in ausreichender Auflösung möglich. Um individuelle Fragen zu Präparaten zu ermöglichen, nutzten wir die *Breakout-Room*-Funktion von ZOOM®. Über die Kontrollübernahme der Dozenten im *Breakout-Room* war es möglich, auf dem Bildschirm der Teilnehmenden einzelne Strukturen einzustellen und zu erläutern. Das Einstellen bestimmter Strukturen ist digital einfacher als am konventionellen Lichtmikroskop ohne Zeiger. Beide Kurse wurden an je 2 aufeinanderfolgenden Tagen durchgeführt. Das Kursprogramm wurde in 4 Blöcke zu je 90 Minuten aufgeteilt.

### Evaluation der Kurse

**Abb. 2:**
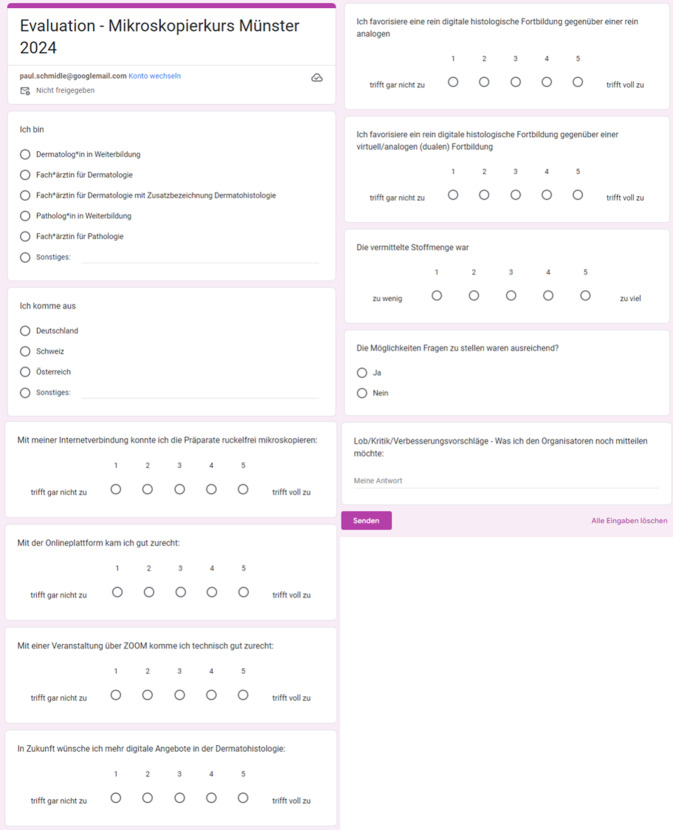
Genutzter Fragebogen zur Evaluation des Einführungs- und Mikroskopierkurses

Mithilfe eines überwiegend standardisierten Fragebogens wurden sowohl die Einführungskurse 2022 und 2024 sowie die Mikroskopierkurse 2021, 2022 und 2024 systematisch evaluiert. Die Teilnehmenden wurden neben der beruflichen Fachbezeichnung auch zu Ausbildungsstand und Herkunftsland befragt. Darüber hinaus wurden Fragen zur technischen Nutzung sowie inhaltliche Fragen zum beigewohnten Kurs gestellt. Hierzu wurde eine 5‑stufige Likert-Skala gewählt von 0 (stimme nicht zu) bis 5 (stimme sehr zu). Zusätzlich gab es eine offene Frage mit Freitextantwort. Die Fragebögen wurden über Google Formulare (Google LLC, Mountain View, Kalifornien, USA) erstellt und von den Teilnehmenden online nach Beendigung des Kurses freiwillig ausgefüllt. Beim Einführungskurs und Mikroskopierkurs wurden die gleichen Fragen gestellt. Der genutzte Fragebogen findet sich in Abb. 2.

### Statistische Auswertung

Im Rahmen des Mikroskopierkurses und des Einführungskurses wurden demografische Variablen sowie Kursinhalte analysiert. Die Datenerhebung erstreckte sich über mehrere Jahre (Mikroskopierkurs: 2021, 2022, 2024; Einführungskurs: 2022, 2024). Der berufliche Hintergrund der Kursteilnehmenden sowie die Items zu spezifischen Kursinhalten wurden deskriptiv untersucht.

Aufgrund der kontinuierlichen Anpassung der Kursinhalte und des Ausbaus digitaler Lehrformate wurden potenzielle zeitliche Trends in den Kursbewertungen mittels Kruskal-Wallis-Tests analysiert. Für den Mikroskopierkurs wurden zusätzlich Dunn’s Post-hoc-Tests durchgeführt, um zu identifizieren, zwischen welchen Erhebungsjahren potenzielle signifikante Unterschiede bestanden. Darüber hinaus wurden im Mikroskopierkurs potenzielle Unterschiede in den Itemantworten zwischen Teilnehmenden aus der Pathologie und der Dermatologie mit Kruskal-Wallis-Tests untersucht.

## Ergebnisse

### Einführungskurs

Die Evaluation des Einführungskurses 2022 wurde von 145 Teilnehmenden ausgefüllt, die des Kurses 2024 von 165. Sowohl den Einführungskurs 2022 wie auch 2024 besuchten mit 76,4 % bzw. 64,8 % vorwiegend Dermatologen in Weiterbildung aus dem deutschsprachigen Raum (> 90 % für beide Jahre).

Im Kurs 2022 konnten 74,8 % der Teilnehmenden die Präparate mit ihrer Internetverbindung ohne technische Probleme, ruckelfrei auf der Plattform mikroskopieren (5/5), 2024 waren es 82,5 %. Während 2022 87,5 % der Teilnehmenden mit der Online-Plattform sehr gut zurechtkamen (5/5), waren es 2024 90,1 %. Auch mit dem Veranstaltungsformat über ZOOM® kamen die meisten Teilnehmenden sehr gut (5/5) zurecht (2022: 87,5 %; 2024: 87,8 %). Etwa drei Viertel der Teilnehmenden wünschte sich zudem für die Zukunft mehr digitale Angebote in der dermatopathologischen Ausbildung (2022: 75 %; 2024: 74,8 %). Stimmte 2022 die Mehrheit der Teilnehmenden (38,5 %) auf die Frage, ob sie eine rein digitale histologische Fortbildung gegenüber einer rein analogen Veranstaltung vorziehen würden, noch „bedingt“ zu (3/5), lag die mehrheitliche Zustimmung 2024 (39,6 %) bereits bei „stimme sehr zu“ (5/5). Insgesamt wurde die vermittelte Stoffmenge sowohl 2022 (65 %) als auch 2024 (57 %) mehrheitlich als gut gewählt (3/5) gewertet.

**Abb. 3:**
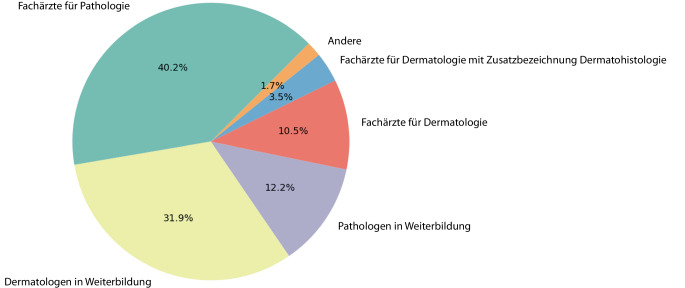
Durchschnittlicher prozentualer Anteil der Teilnehmenden nach Ausbildungsstand und Fachrichtung im Mikroskopierkurs über die Jahre 2021, 2022 und 2024

### Mikroskopierkurs

Die Evaluation des Mikroskopierkurses 2021 wurde von 55 Teilnehmenden, 2022 von 69 und 2024 von 106 ausgefüllt. Anders als die Einführungskurse, die vorwiegend von Dermatologen in Weiterbildungen besucht wurden, wurden die Mikroskopierkurse 2021 (34,5 %) und 2022 (36,8 %) fast zu gleichen Teilen, 2024 sogar mehrheitlich (45,3 %), von Fachärzten für Pathologie besucht, ebenfalls zu > 90 % aus dem deutschsprachigen Raum. Nimmt man noch die Pathologen in Weiterbildung hinzu, waren über die Jahre mehr als die Hälfte der Teilnehmenden (52,4 %) fertige oder angehende Pathologen (Abb. 3).

Im Kurs 2021, dem ersten rein digital durchgeführten Kurs, konnten 88,9 % der Teilnehmenden die Präparate ohne technische Probleme, ruckelfrei mikroskopieren (5/5), 2022 waren es 85,5 %, 2024 86,8 %. Während 2021 bereits 88,9 % der Teilnehmenden mit der Online-Plattform sehr gut zurechtkamen (5/5), waren es 2022 und 2024 sogar 97 % bzw. 95,2 %. Im Jahr 2021 gaben zudem 88,9 % der Befragten an, dass die Plattform intuitiv und selbsterklärend sei (5/5), 92,7 % der Teilnehmenden konnten 2021 zudem durch die Annotationen die Präparate einfach und selbstständig mikroskopieren (5/5). Auch mit dem Veranstaltungsformat über Zoom® kamen die allermeisten der Teilnehmenden sehr gut (5/5) zurecht (2021: 89,1 %; 2022: 88,4 %, 2024: 92,5 %). Über drei Viertel der Teilnehmenden wünschten sich zudem für die Zukunft mehr digitale Angebote in der dermatopathologischen Ausbildung (2021: 85,2 %; 2022: 75,4 %; 2024: 83,3 %). Stimmte 2021 die Mehrheit der Teilnehmenden (40 %) auf die Frage, ob sie eine rein digitale histologische Fortbildung gegenüber einer rein analogen Veranstaltung vorziehen würden, noch „bedingt“ zu (3/5), lag die mehrheitliche Zustimmung 2022 knapp (31,7 %) und 2024 bereits sogar deutlich (46,2 %) bei „stimme sehr zu“ (5/5). Auf die Frage, ob sie eine rein digitale Fortbildung gegenüber einer hybriden Veranstaltung bevorzugen würden, antwortete jedoch sowohl 2021 (37,7 %) als auch 2022 (37,7 %) und 2024 (34,3 %) die Mehrheit mit „stimme bedingt zu“ (3/5). Insgesamt wurde die vermittelte Stoffmenge sowohl 2021 (79,6 %) als auch 2024 (70,8 %) mehrheitlich als gut gewählt (3/5) gewertet. Im Jahr 2022 wurde die vermittelte Stoffmenge im Vergleich zu 2021 jedoch eher als zu hoch eingeschätzt (*p* = 0,002).

Es fanden sich in der Subgruppenanalyse kleine Unterschiede zwischen den Fachbereichen. Im Mikroskopierkurs fanden, gemittelt über die Jahre, die Dermatologen die Annotationen etwas hilfreicher als die Pathologen (5/5 vs. 4,8/5), präferierten die Dermatologen noch stärker rein digitale Fortbildungen (4,0/5 vs. 3,7/5) und bewerteten die Pathologen die vermittelte Stoffmenge eher als zu hoch (3,2/5 vs. 3,4/5).

Insgesamt wurden beide Kurse sehr gut bewertet (4,5/5), ebenso gibt es bei beiden Kursen eine klare Präferenz hin zu mehr rein digitaler Lehre.

## Diskussion

Die Digitalisierung schreitet auch in der Pathologie rasch voran. Moderne Scanner machen es mittlerweile möglich, die Glasobjektträger innerhalb von Minuten zu digitalisieren [14]. Diese WSI können über Cloud-basierte Online-Plattformen rasch geteilt und einem größeren Publikum Browser-basiert zur Verfügung gestellt werden. Dies eröffnet in der Ausbildung viele neue Möglichkeiten. Die Kombination aus online durchgeführter Videokonferenz unter Nutzung der Cloud-basierten-Plattformen macht es möglich, auch komplexere pathologische Lehrinhalte online zu vermitteln [15]. Die Online-Formate der Münsteraner Dermatopathologiekurse sind hierfür ein Beleg.

Auf technischer Seite hatten die Kursteilnehmenden von Beginn an nur wenige Probleme. Dies lässt sich damit erklären, dass die Nutzung von Videokonferenzen seit der COVID-19-Pandemie bei vielen von uns zum Arbeitsalltag gehört. Die genutzte ZOOM®-Software überzeugte dabei v. a. durch die schnelle, ruckelfreie Übertragung der Bilder während der Demonstration der WSI durch die Dozenten. Die Online-Pathologieplattform SmartZoom® [16] bietet eine einfache, intuitive Bedienung bei übersichtlichem Aufbau. Sie ist bereits an vielen verschiedenen Universitäten für den Studierendenunterricht in der Anatomie und Pathologie im Einsatz. Eine alternative Plattform, die v. a. in den USA genutzt wird, ist PathPresenter® [17]. Hierüber hat die Internationale Gesellschaft für Dermatopathologie (ISDP) bereits eine Jahrestagung online ausgerichtet. Auch SmartZoom® bietet weitere Tools wie einen speziellen „Hausaufgaben“- und Fragen-Modus, wodurch Prüfungen realisiert werden können. Die Prüfungspräparate der „International Board Certification in Dermatopathology“ sollen zukünftig auch digital über eine Plattform mikroskopiert werden.

Digital angebotene Kurse sind mittlerweile aber nicht nur technisch gut umsetzbar, sie bringen auch auf organisatorischer Ebene viele Vorteile mit sich – für Veranstalter wie Teilnehmende. Aufseiten der Veranstalter müssen die Glasobjektträger beispielsweise nicht mehr vervielfältigt werden. Digital wird somit immer die identische Schnittstufe mit denselben Annotationen für alle Teilnehmenden zur Verfügung gestellt. Die Organisation von Räumlichkeiten mit Mikroskopen entfällt. Die Teilnehmenden verlieren keine Zeit für die An- und Abreise und sparen Reise- und Übernachtungskosten.

Die Evaluation bestätigte unseren Eindruck der letzten Jahre, dass gerade die Vermittlung detaillierter, visueller Informationen rein digital am Bildschirm gut funktioniert. Insbesondere die Möglichkeit, durch die gezielte Anordnung von Annotationen die Teilnehmenden systematisch durch ein histologisches Präparat zu leiten und auf spezielle Clues hinzuweisen, erscheint didaktisch besonders wertvoll. Die Teilnehmenden können hierdurch dem erfahrenen Befunder Schritt für Schritt durch einen Schnitt folgen, diesen somit praktisch nachahmen, was auch ein effektives Eigenstudium möglich macht. Der zunehmende Wunsch nach einem größeren digitalen Lehrangebot, der sich in fortwährend steigenden Zustimmungswerten über die Jahre ab 2021 aus den Evaluationen herauslesen lässt, zeigt, wie sehr die Teilnehmenden mittlerweile die zuvor bereits erwähnten Vorteile von Onlinekursen schätzen.

Weitergedacht werden durch Onlineangebote Lehrinhalte überhaupt erst zugänglich für bestimmte Zielgruppen, beispielsweise aus benachteiligten Regionen der Welt. Auch entfällt die ansonsten limitierte Teilnehmerzahl von Kursen, und es können gleichzeitig deutlich mehr Interessierte geschult und ausgebildet werden.

**Abb. 4:**
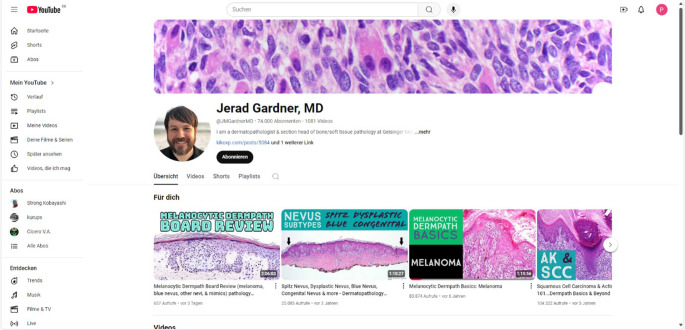
YouTube®-Kanal von Jerad Gardner mit kostenlos zugänglichen dermatopathologischen Lehrvideos

Neben dem Münsteraner Dermatopathologiekurs finden sich auch viele weitere digitale Ausbildungsmöglichkeiten für Dermatopathologen. Online finden sich kostenpflichtige Lehrangebote wie Dermpedia [18], teilweise bieten Kollegen aber auch kostenloses Ausbildungsmaterial an. Ein Beispiel hierfür sind die frei zugänglichen Lehrvideos auf YouTube® von Jerad Gardner ([19]; Abb. 4) oder Arnaud de la Fourchardière [20], die in anschaulicher, äußerst informativer Art und Weise Dermatopathologie erklären. Auch in Social-Media-Gruppen wie etwa der von Philip McKee gegründeten Facebook-Gruppe [21] oder auf KiKo [22], einer Online-Plattform der nächsten Generation, die soziale Medien mit digitaler Pathologie kombiniert, wird mittlerweile dermatopathologisches Wissen geteilt und ausgetauscht.

Auch die Arbeitsgemeinschaft Dermatologische Histologie (ADH) stellt ihren Mitgliedern seit einiger Zeit die Schnittseminare ihrer Veranstaltungen online zur Verfügung [5, 23]. Und auch klinisch-pathologische Konferenzen wie sie an der Hautklinik des Universitätsklinikums Münster oder von Prof. Cerroni aus der Universitätsklinik für Dermatologie und Venerologie der Universität Graz angeboten werden, finden aktuell im digitalen Raum statt, um ein größeres Publikum zu erreichen. Ebendiesem Ziel folgt auch der Plan, die Münsteraner Dermatopathologiekurse im Herbst 2025 erstmals auf Englisch stattfinden zu lassen. Hierdurch soll insbesondere ein Publikum erreicht werden, das sonst keinen Zugang zur dermatopathologischen Ausbildung hat.

Bei allem Enthusiasmus über digitale Lehrangebote soll nicht unerwähnt bleiben, dass bei rein digitalen Formaten der persönliche Austausch zwischen den Teilnehmenden wegfällt. Jeder, der einmal an einem Kongress in Präsenz teilgenommen hat, wird bestätigen können, dass gerade beim Small-Talk in den Pausen Kontakte geknüpft werden und die besten Ideen und Projekte entstehen. Diese Art der Interaktion konnte bisher noch mit keinem Format optimal in den digitalen Raum übertragen werden. Außerdem soll nicht unerwähnt bleiben, dass die analoge Mikroskopie in manchen Aspekten dem digitalen Mikroskopieren noch überlegen ist. Bestes Beispiel hierfür ist das einfache Nachjustieren am Feintrieb, das speziell bei der Erkennung dreidimensionaler, in die Bildebene ragender Strukturen helfen kann, und digital nur mit höherem Aufwand nachgestellt werden kann.

Zusammenfassend bleibt festzuhalten, dass die digitale Ausbildung junger Dermatopathologen viele Vorteile bietet. Die durchweg positive Evaluation der Münsteraner Dermatopathologiekurse zeigt, dass der Bedarf an digitaler Aus- und Weiterbildung gegeben ist – und das nicht nur im deutschsprachigen Raum, sondern auch international. Digitale Lehrangebote werden sich als zusätzlicher Eckpfeiler in der der dermatopathologischen Ausbildung durchsetzen – davon sind die Autoren dieses Beitrags überzeugt.

## Fazit für die Praxis

- Digitale Lehrformate können auch in der Dermatopathologie in Zukunft die Ausbildung ergänzen.
- Technisch sind sie bereits heute gut umsetzbar.
- Viele wünschen sich in Zukunft noch mehr digitale Lehrangebote in der Dermatopathologie.
- Bei digitalen Angeboten leidet jedoch der persönliche Austausch.
- Eine Kombination aus verschiedenen Präsenz- wie digitalen Formaten scheint für die Zukunft daher ein sinnvoller Weg zu sein.

## Data Availability

Die Daten, die die Ergebnisse dieser Studie untermauern, sind auf begründete Anfrage beim Korrespondenzautor erhältlich.
